# Relationship between cardiac deformation parameters measured by cardiovascular magnetic resonance and aerobic fitness in endurance athletes

**DOI:** 10.1186/s12968-016-0266-x

**Published:** 2016-08-17

**Authors:** Peter P. Swoboda, Bara Erhayiem, Adam K. McDiarmid, Rosalind E. Lancaster, Gemma K. Lyall, Laura E. Dobson, David P. Ripley, Tarique A. Musa, Pankaj Garg, Carrie Ferguson, John P. Greenwood, Sven Plein

**Affiliations:** 1 Multidisciplinary Cardiovascular Research Centre (MCRC) and Leeds Institute of Cardiovascular and Metabolic Medicine, University of Leeds, Clarendon Way, Leeds, LS2 9JT UK; 2 Multidisciplinary Cardiovascular Research Centre (MCRC) and School of Biomedical Sciences, University of Leeds, Clarendon Way, Leeds, LS2 9JT UK

**Keywords:** Cardiovascular magnetic resonance, Tissue tagging, Feature tracking, Athlete, Aerobic capacity, Lactate threshold

## Abstract

**Background:**

Athletic training leads to remodelling of both left and right ventricles with increased myocardial mass and cavity dilatation. Whether changes in cardiac strain parameters occur in response to training is less well established. In this study we investigated the relationship in trained athletes between cardiovascular magnetic resonance (CMR) derived strain parameters of cardiac function and fitness.

**Methods:**

Thirty five endurance athletes and 35 age and sex matched controls underwent CMR at 3.0 T including cine imaging in multiple planes and tissue tagging by spatial modulation of magnetization (SPAMM). CMR data were analysed quantitatively reporting circumferential strain and torsion from tagged images and left and right ventricular longitudinal strain from feature tracking of cine images. Athletes performed a maximal ramp-incremental exercise test to determine the lactate threshold (LT) and maximal oxygen uptake (V̇O_2max_).

**Results:**

LV circumferential strain at all levels, LV twist and torsion, LV late diastolic longitudinal strain rate, RV peak longitudinal strain and RV early and late diastolic longitudinal strain rate were all lower in athletes than controls. On multivariable linear regression only LV torsion (beta = −0.37, *P* = 0.03) had a significant association with LT. Only RV longitudinal late diastolic strain rate (beta = −0.35, *P* = 0.03) had a significant association with V̇O_2max_.

**Conclusions:**

This cohort of endurance athletes had lower LV circumferential strain, LV torsion and biventricular diastolic strain rates than controls. Increased LT, which is a major determinant of performance in endurance athletes, was associated with decreased LV torsion. Further work is needed to understand the mechanisms by which this occurs.

## Background

It is well recognised that athletic training leads to ventricular remodelling, specifically increases in left and right ventricular end diastolic volume (LVEDV & RVEDV) and left ventricular mass (LVM) [1, 2]. These structural changes are most frequently seen in athletes who undergo prolonged periods of endurance training [3].

Although less well established, there is also evidence for changes in functional parameters in the hearts of trained athletes. Endurance athletes have reduced ejection fraction, circumferential and longitudinal strain of both the left and right ventricles compared to healthy controls [4–6]. The heart has a complex twisting motion where the base rotates clockwise in early systole and the apex rotates anticlockwise in later systole. These opposing directions of rotation at the apex and base generate maximal torsional force at end systole [7]. It has been reported that athletes have decreased LV twist and torsion when compared to controls [5].

Both strain and torsion parameters can be measured using cardiovascular magnetic resonance (CMR) tissue tagging techniques [8]. CMR tagging is considered to be the gold standard for measurement of myocardial strain and torsion [9, 10]. More recently, post-processing feature tracking of cine images has been proposed for quantification of strain without the need for acquisition of tagged CMR data [11]. Previous studies have shown good agreement between strain parameters derived from feature tracking and tissue tagging [11].

Cardiopulmonary exercise testing (CPX) with breath-by-breath measurement of gas exchange responses is an established method for assessment of whole-body exercise tolerance, and key parameters of aerobic function: the lactate threshold (LT) and the maximal pulmonary oxygen uptake (V̇O_2max_) [12]. This information gives an accurate and reproducible measure of the integrated capacity of the respiratory, cardiovascular, and neuromuscular systems, and is frequently used to quantitatively assess aerobic capacity and training status [13]. Previous studies have demonstrated a clear correlation between LV remodelling and V̇O_2max_ in endurance athletes [14–16].

In this study we investigated the relationship between strain-derived parameters of cardiac function and CPX-derived performance parameters that has not previously been investigated. We hypothesised that strain parameters measured at rest would be lower in athletes than in controls and lowest in athletes with the highest V̇O_2max_. We also planned to specifically investigate if any strain parameters were associated with LT.

## Methods

### Enrolment recruitment

Thirty five endurance athletes were recruited from local sporting clubs. They all trained more than 6 h a week and competed regularly at local, national or international level. Exclusion criteria were any medical illness or contraindication to CMR. Thirty five controls who exercised less than 3 h a week were also recruited and prospectively matched to the athletes for age and gender. No athletes or controls had any medical condition or took any regular medication.

### CMR protocol

CMR was performed on a dedicated cardiovascular 3 Tesla Philips Achieva system equipped with a 32 channel coil and MultiTransmit® technology. Data was acquired during breath-holding at end expiration. From scout CMR images, the left ventricular long and short axes were determined.

Cine images covering the entire heart in the LV short axis plane were acquired (balanced steady state free precession (SSFP), spatial resolution 1.2×1.2×10mm^3^, 30 cardiac phases TR/TE 2.6/1.3 ms, flip angle 40°, field of view 300–420 mm, typical temporal resolution 39 ms) and in orthogonal long-axis planes. Then axial cine images planned to cover the right ventricle were acquired (balanced SSFP, spatial resolution 1.2 × 1.2 × 6 mm^3^, 30 cardiac phases TR/TE 2.6/1.3 ms, flip angle 40°, field of view 300–420 mm).

Tissue tagging by spatial modulation of magnetization (SPAMM) (spatial resolution 1.51 × 1.57 × 10 mm^3^, tag separation 7 mm, ≥18 phases, typical TR/TE 5.8/3.5 ms, flip angle 10°, typical temporal resolution 55 ms) was acquired in the three short axis slices acquired at the apex, mid-ventricle, and base. Slices were positioned using the highly reproducible “3 of 5 technique” [17].

### Image analysis

CMR data were analysed quantitatively using commercially available software (CVI42, Circle Cardiovascular Imaging Inc. Calgary, Canada and inTag v1.0, CREATIS lab, Lyon, France). Epicardial and endocardial borders were traced on the LV and RV cine stack at end-diastole and end-systole to calculate end diastolic volume (LVEDV), end systolic volume (ESV), stroke volume (SV), ejection fraction (EF) and LV mass. Volumes were indexed to body surface area (BSA) calculated using the Mosteller equation.

For tagging analysis endocardial and epicardial contours were drawn on the short axis SPAMM sequences using a semi-automated process. Peak circumferential LV strain was measured for the three slices at apex, mid-ventricle, and base. Peak systolic and both early and late diastolic LV strain rates were measured from the mid-ventricular slice. Strain was measured in the mid-myocardial layer which has previously been reported to be the most reproducible [18]. LV twist was calculated by subtracting the basal from apical rotation. Basal and apical radius was calculated from cine images in diastole at the same slice location as the tagged images. The equation used to determine torsion was [10]:

$$ Torsion=\frac{Peak\kern0.5em  Twist\kern0.5em \times \kern0.5em \left( Apical\kern0.5em  Radius+ Basal\kern0.5em  Radius\right)}{2\kern0.5em \times \kern0.5em  Apex\kern0.5em to\kern0.5em  Base\kern0.5em  length} $$

For feature tracking analysis endocardial and epicardial contours were drawn on a long axis 4 chamber cine using a semi-automated process. Peak longitudinal strain, systolic strain rate (SSR), early and late diastolic strain rates (EDSR and LDSR) were measured for both the LV & RV. LDSR was defined as peak rate during atrial contraction. We have used feature tracking rather than SPAMM for the analysis of longitudinal strain. However, tissue tagging is hampered by a lower temporal resolution than cine imaging and tag fading during diastole. As we specifically wanted to examine longitudinal strain rates in diastole we therefore chose to use feature tracking for this while using SPAMM tagging for assessment of circumferential strain parameters.

### Exercise protocol

A ramp-incremental test (20–30 W/min) to the limit of tolerance was performed on a cycle ergometer (Excalibur Sport, Lode BV, Groningen, the Netherlands), with breath-by-breath pulmonary gas exchange measured throughout (Cardio2, Medgraphics, Medical Graphics Corporation, St Paul, MN, USA). A 12-lead ECG was also monitored throughout this test, with heart rate determined from the R-R interval. LT was estimated using standard ventilatory and pulmonary gas-exchange criteria (REF), and V̇O_2max_ determined as the highest 12-breath mean. An additional step-exercise test performed to the limit of tolerance confirmed that V̇O_2max_ was attained in all participants [19].

### Statistical analysis

Statistical analysis was performed using IBM SPSS® Statistics 20.0 (IBM Corp., Armonk, NY). Continuous variables were expressed as means ± SD. Categorical variables were expressed as *N* (%). Shapiro-Wilk test was used to test normality and unpaired t-tests and Mann Whitney *U* test used to compare athletes and controls. Pearson’s coefficient was used to measure correlation between exercise and CMR parameters. Univariable analyses were performed to identify predictors of LT and V̇O_2max_. Variables with a probability value <0.1 in the univariable analysis were included in a multivariable linear regression analysis. The standardised coefficient (beta) is reported. *P* < 0.05 was considered statistically significant.

## Results

### Study participant demographics and characteristics

Of 35 athletes 7 were runners, 15 cyclists and 13 triathletes. The athletes trained 11.5 ± 3.7 h per week and all had trained >6 h per week for 8.4 ± 6.0 years. Mean ramp duration was 772 ± 93 s reaching a peak work rate of 370 ± 64 W. HR rose from 55 ± 7 beats/min at rest to 182 ± 10 beats/min at peak exercise. Mean LT was 2.60 ± 0.57 l/min, 36.5 ± 6.7 ml/min/kg (normalised to body weight) or 62.1 ± 8.0 % V̇O_2max_. Mean V̇O_2max_ was 4.2 ± 0.80 l/min, 58.9 ± 8.2 ml/min/kg or 160.0 ± 18.8 % of predicted V̇O_2max_ [20].

Athletes and controls were prospectively matched for age and gender (Table 1). BMI and resting heart rate were lower in athletes than controls (*P* = 0.001 and *P* < 0.001 respectively).

**Table 1:** Subject characteristics

	Athlete	Control	*P* value
Age	31.3 ± 7.6	30.6 ± 8.5	0.72
Male, %	27 (77)	27 (77)	1.0
Height, cm	178.7 ± 8.7	176.5 ± 8.2	0.29
Weight, kg	71.4 ± 9.9	77.0 ± 14.8	0.07
BMI, kg/m^2^	22.3 ± 1.9	24.5 ± 3.3	0.001
HR	55.0 ± 6.5	65.1 ± 8.7	<0.001
SBP, mmHg	118.8 ± 8.7	114.7 ± 10.6	0.16
DBP, mmHg	71.0 ± 9.2	59.2 ± 10.6	<0.001

### CMR findings

**Fig. 1:**
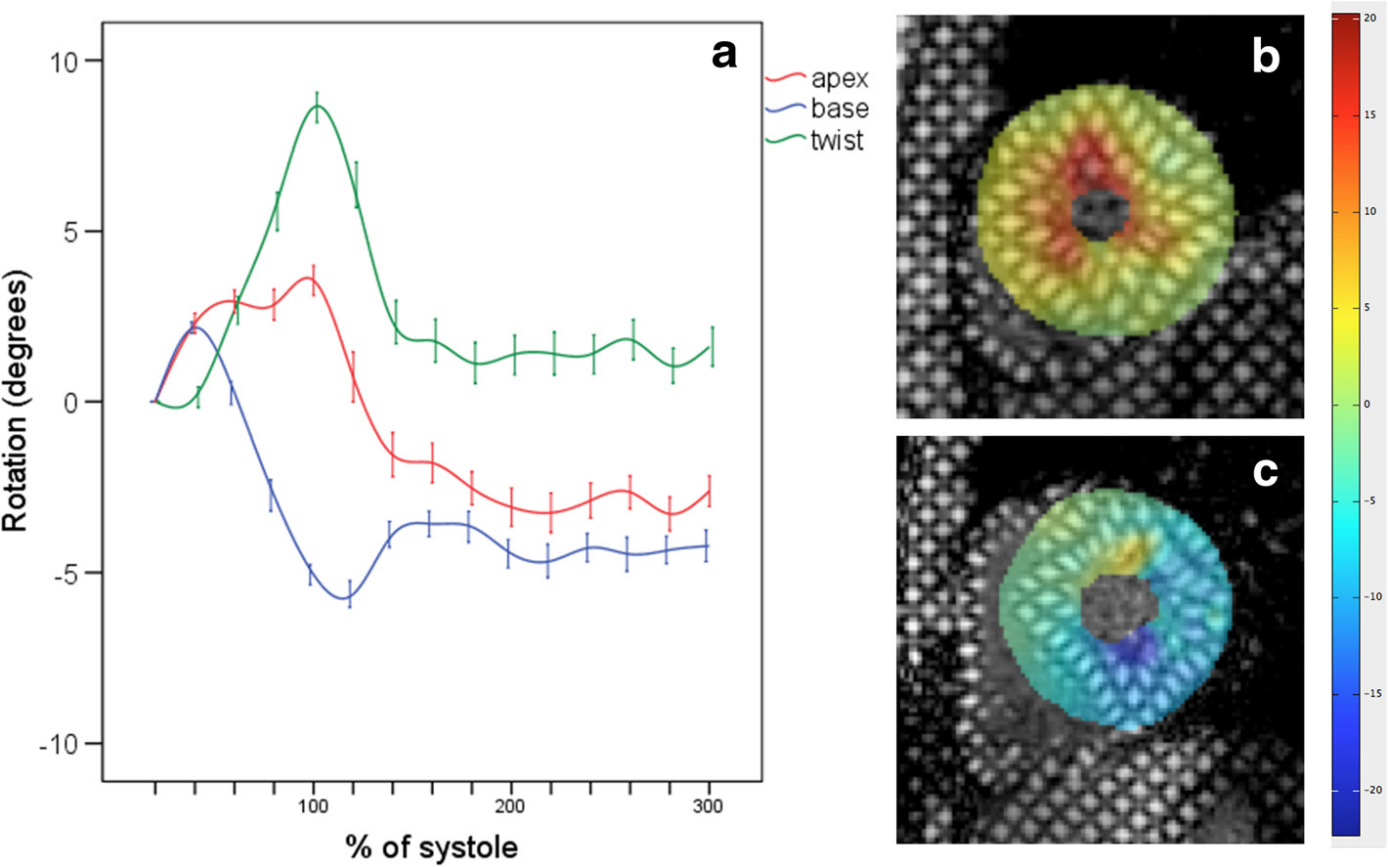
Average apical rotation (red), basal (blue) rotation and twist (green) of the left ventricle of 35 endurance athletes (**a**). Each point represents mean rotation/twist and time in the cardiac cycle corrected to end-systole, error bars represent standard error of mean rotation/twist. Tagged images of anticlockwise apical (**a**) systolic rotation (yellow and red) and clockwise basal (**c**) rotation (green and blue)

**Fig. 2:**
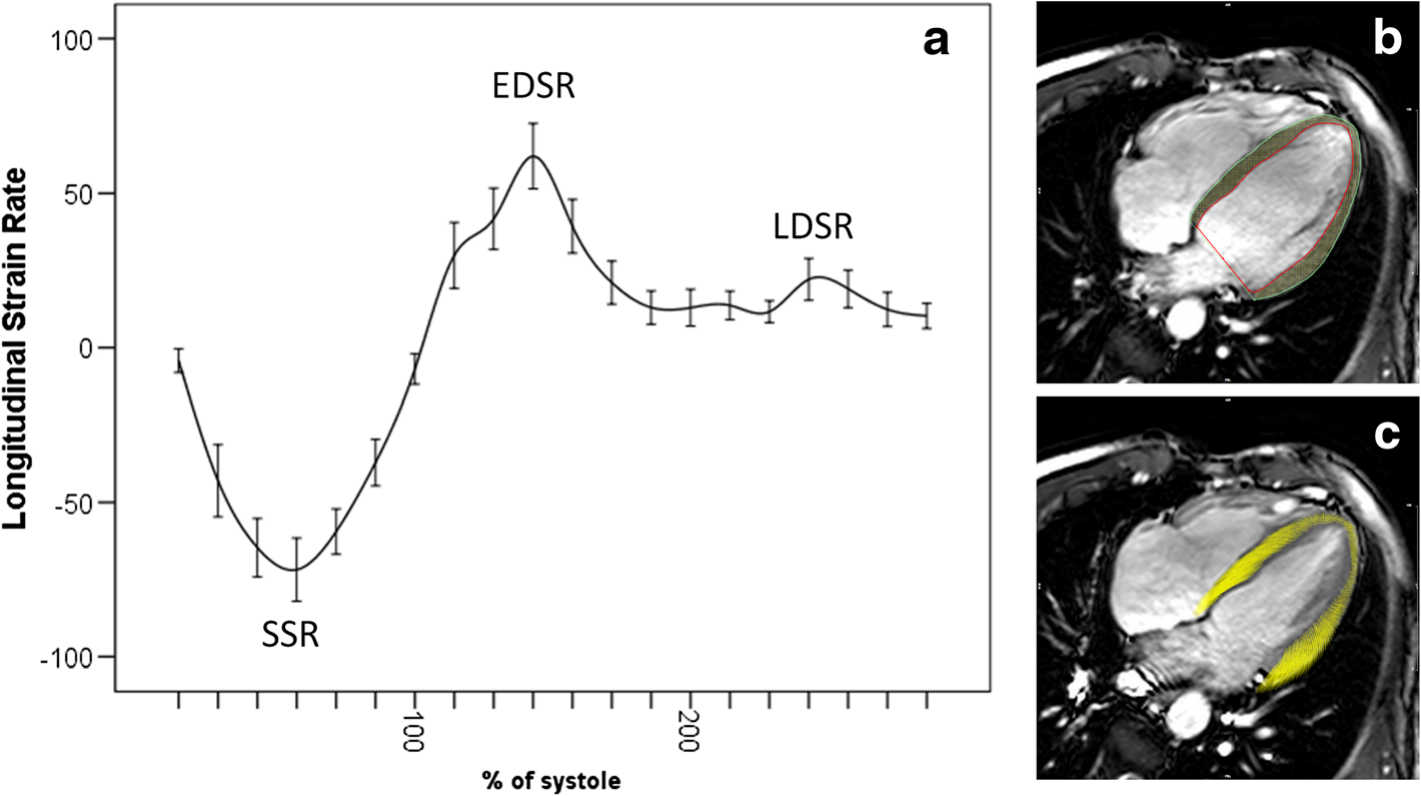
Average LV longitudinal strain rate from 35 endurance athletes (**a**). Each point represents mean longitudinal strain rate at each point in the cardiac cycle corrected to end-systole, error bars represent 95 % confidence interval of mean strain. Peak systolic strain rate (SSR), early diastolic strain rate (EDSR) and late diastolic strain rate (LDSR). SSFP cine image at end diastole showing manually drawn endocardial and epicardial contours (**b**). Feature tracked end systolic image (**c**)

LV volumes for athletes and controls are shown in Table 2. LVEDV, LVM and RVEDV indexed to BSA were greater in athletes than controls. LVEF was lower in athletes than controls (*P* = 0.04) but there was no difference in RVEF (*P* = 0.27). Strain parameters are shown in Table 3. LV circumferential strain at all levels, LV twist and torsion (Fig. 1), LV longitudinal LDSR (Fig. 2), RV peak longitudinal strain and RV EDSR and LDSR were all lower in athletes than controls.

**Table 2:** CMR measured volumetric parameters

	Athlete	Control	*P* value
Left ventricle			
EDV, ml	217.1 ± 34.8	176.5 ± 34.8	<0.001
EDVI, ml/m^2^	115.4 ± 14.2	90.8 ± 12.9	<0.001
ESV, ml	96.1 ± 18.7	74.7 ± 18.7	<0.001
Ejection Fraction, %	55.7 ± 4.5	57.9 ± 4.1	0.04
LVM, g	127.9 ± 24.6	100.5 ± 23.4	<0.001
LVMI, g/m^2^	67.8 ± 9.9	51.5 ± 9.1	<0.001
LVM/EDV, g/ml	0.59 ± 0.07	0.57 ± 0.08	0.25
Right ventricle			
EDV, ml	219.7 ± 37.2	204.8 ± 50.1	0.16
EDVI, ml/m^2^	116.8 ± 15.8	105.1 ± 19.7	0.01
ESV, ml	104.2 ± 22.7	99.5 ± 27.5	0.44
Ejection Fraction, %	52.8 ± 4.7	51.6 ± 3.7	0.27

**Table 3:** CMR measured strain parameters

	Athlete	Control	*P* value
LV Circumferential Strain			
Apex, %	18.4 ± 5.2	23.4 ± 4.9	<0.001
Mid LV, %	19.6 ± 3.9	21.5 ± 2.5	0.02
Base, %	17.0 ± 4.0	20.5 ± 2.5	<0.001
Systolic SR, %/s	115.3 ± 12.8	116.6 ± 10.0	0.66
Early diastolic SR,%/s	50.8 ± 16.4	51.0 ± 16.0	0.95
Late diastolic SR, %/s	140.1 ± 40.7	151.4 ± 40.3	0.27
Torsion			
LV twist, ^o^	9.7 ± 3.6	13.3 ± 3.8	<0.001
LV torsion, ^o^	8.8 ± 3.0	11.9 ± 3.1	<0.001
LV twist rate, ^o^/s	63.2 ± 18.9	72.4 ± 27.8	0.048
LV untwist rate, ^o^/s	88.1 ± 25.5	101.8 ± 34.5	0.07
LV Longitudinal Strain			
Peak, %	17.1 ± 2.8	17.7 ± 2.3	0.30
SSR, %/s	101.6 ± 29.6	103.2 ± 19.8	0.29
EDSR, %/s	90.6 ± 32.4	102.4 ± 31.7	0.13
LDSR, %/s	41.7 ± 15.6	57.3 ± 19.6	<0.001
RV Longitudinal Strain			
Peak, %	19.8 ± 3.7	22.6 ± 3.4	0.002
SSR, %/s	137.7 ± 49.9	138.4 ± 37.0	0.50
EDSR, %/s	108.6 ± 32.1	124.6 ± 32.9	0.03
LDSR, %/s	69.2 ± 40.2	89.5 ± 42.4	0.02

In athletes, there were no significant correlations between left ventricle mass indexed to BSA (LVMI), left ventricle end diastolic volume indexed to BSA (LVEDVI) and LVM/EDV and LV twist (*P* = 0.20, 0.85 and 0.21 respectively). LV torsion had a trend to correlation with LVMI (*R* = −0.34, *P* = 0.05) but there were no significant associations with LVEDVI or LVM/EDV (*P* = 0.61 and 0.08 respectively).

### Relationship between CPX and functional CMR parameters in athletes

**Fig. 3:**
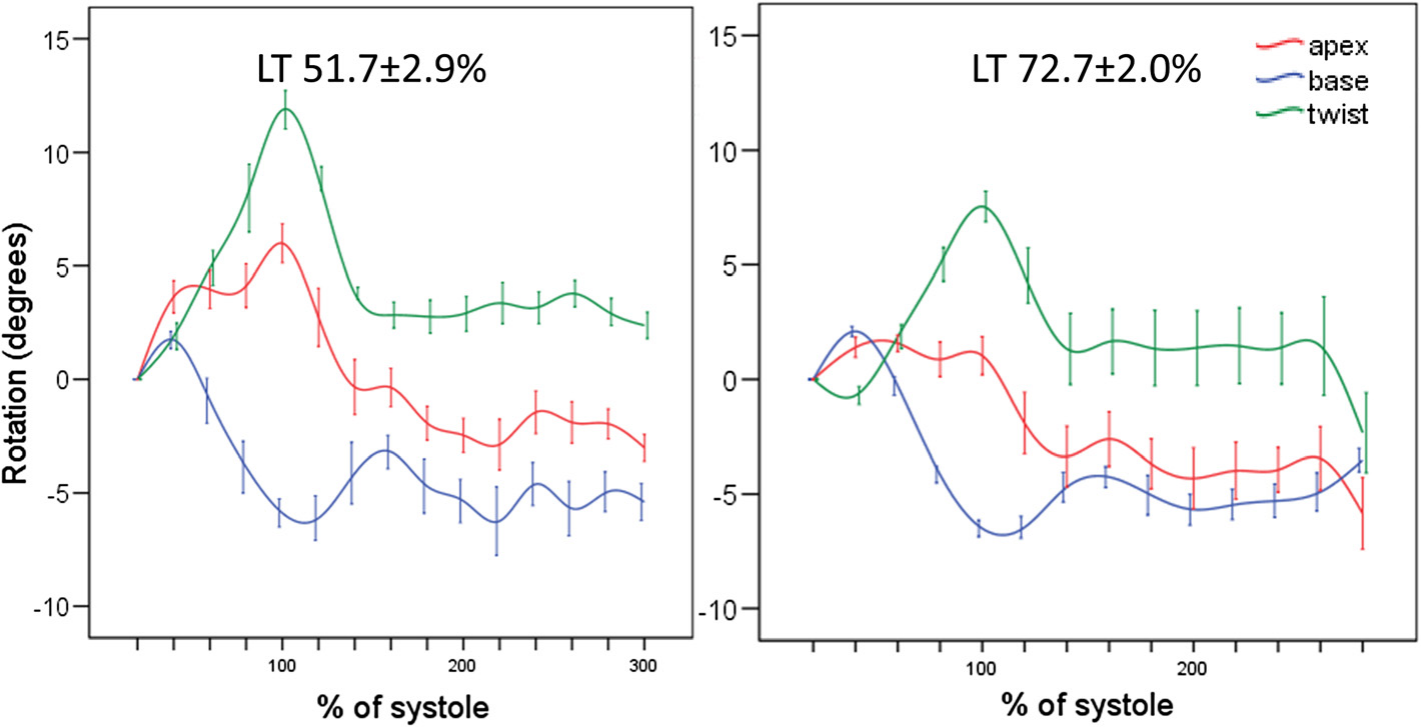
Plots showing the mean ± standard error of rotation/twist of the 8 athletes in the lowest quartile of LT (left) and 8 in the highest quartile of LT (right). Peak twist was lower in athletes in the highest quartile of LT (8.5 ± 2.9 vs. 12.9 ± 2.6° *P* = 0.008) which was secondary to a loss of apical rotation (1.0 ± 3.3 vs. 6.0 ± 3.1° *P* < 0.001). Basal rotation was not different between the groups 6.5 ± 1.4 vs. 6.1 ± 2.6° *P* = 0.75

The only significant correlations with LT (%V̇O_2max_) were with torsion parameters. Peak twist (*r* = −0.45, *P* = 0.01), peak torsion (*r* = −0.36, *P* = 0.04) and twist rate (*r* = −0.38, *P* = 0.03) all had a significant correlation with LT. There was no significant correlation between LT and any demographic, volumetric measurement (of those listed in Table 2) or other strain parameter. The decrease in LV twist and torsion was secondary to decreased apical rotation in the athletes with the highest LT (Fig. 3).

There was a correlation between V̇O_2max_ (normalised to weight) and both LVMI (*r* = 0.59, *P* < 0.001) and LVEDVI (*r* = 0.47, *P* = 0.01) [14, 21]. There was also a trend to correlation between RVEDVI and V̇O_2max_ (*r* = 0.33, *P* = 0.05). No LV strain parameters had a significant correlation with V̇O_2max_. RV longitudinal SSR (*r* = −0.33, *P* = 0.05) and RV LDSR (*r* = −0.38, *P* = 0.02) both correlated with V̇O_2max_.

### Regression analysis

On univariable linear regression of the parameters shown in Table 4 only LV torsion and sex were associated with LT. On multivariable linear regression only LV torsion (beta = −0.37, *P* = 0.03) had a significant association with LT.

**Table 4:** Univariable and multivariable linear regression analysis of factors with a significant association with lactate threshold

	Univariable		Multivariable	
	Beta	*P* value	Beta	*P* value
Age	−0.07	0.70		
Sex	0.29	0.09	0.29	0.08
LVMI, g/m^2^	−0.01	0.98		
LVEDVI, ml/ m^2^	0.15	0.39		
LVEF, %	−0.43	0.81		
RVEF, %	0.10	0.56		
RVEDI, ml/ m^2^	0.26	0.14		
Apex circumferential strain, %	0.15	0.38		
Mid LV circumferential strain, %	−0.11	0.55		
Base circumferential strain, %	−0.05	0.77		
LV torsion, ^o^	−0.36	0.04	−0.37	0.03
LV longitudinal strain, %	0.09	0.61		
LV longitudinal SSR, %/s	0.15	0.40		
LV longitudinal EDSR, %/s	−0.23	0.19		
LV longitudinal LDSR, %/s	−0.01	0.95		
RV longitudinal strain, %	−0.01	0.97		
RV longitudinal SSR, %/s	0.04	0.84		
RV longitudinal EDSR, %/s	−0.01	0.97		
RV longitudinal LDSR, %/s	−0.22	0.20		

On univariable linear regression of the parameters shown in Table 5 age, sex, LVEDVI, LVMI, RVEDVI, RV longitudinal strain, RV longitudinal SSR and LDSR were associated with V̇O_2max_. On multivariable linear regression only RV longitudinal LDSR (beta = −0.39, *P* = 0.03) had a significant association with V̇O_2max_.

**Table 5:** Univariable and multivariable linear regression analysis of factors with a significant association with V̇O_2max_

	Univariable		Multivariable	
	Beta	*P* value	Beta	*P* value
Age	−0.37	0.04	−0.26	0.10
Sex	0.40	0.02	−0.30	0.14
LVMI, g/m^2^	0.59	<0.001	0.19	0.37
LVEDVI, ml/ m^2^	0.47	0.01	0.16	0.48
LVEF, %	−0.12	0.50		
RVEF, %	−0.52	0.77		
RVEDI, ml/m^2^	0.33	0.05	0.06	0.78
Apex circumferential strain, %	0.27	0.11		
Mid LV circumferential strain, %	0.18	0.92		
Base circumferential strain, %	−0.17	0.33		
LV torsion, ^o^	−0.21	0.24		
LV longitudinal strain, %	0.17	0.32		
LV longitudinal SSR, %/s	0.04	0.80		
LV longitudinal EDSR, %/s	−0.04	0.81		
LV longitudinal LDSR, %/s	−0.14	0.41		
RV longitudinal strain, %	0.30	0.08	−0.15	0.45
RV longitudinal SSR, %/s	−0.33	0.05	−0.24	0.14
RV longitudinal EDSR, %/s	−0.01	0.96		
RV longitudinal LDSR, %/s	−0.38	0.02	−0.35	0.03

## Discussion

We have carried out comprehensive cardiac functional assessment of 35 endurance athletes from a broad spectrum of event type, age and athletic ability (range 123–206 % predicted V̇O_2max_). This diverse, but well characterised group has allowed us to investigate specifically the ventricular strain parameters that have a relationship with key parameters of aerobic function and exercise capacity, namely LT and V̇O_2max_ [22].

### The relationship between LV torsion and lactate threshold

The most striking finding was the inverse linear correlation between both LV twist and torsion, and LT. On multivariable linear regression no other factors significantly influenced LT. To our knowledge this is the first time a significant association between a cardiac structural or functional parameter and LT (key parameter of aerobic function) has been reported. Furthermore we have found that in athletes with the highest LT the decrease in torsion is secondary to decreased apical rotation.

We have reported with high statistical significance that LV torsion was lower in endurance athletes than controls. Previous CMR tagging studies have been small and insufficiently powered and therefore unable to report a difference in baseline torsion parameters between endurance athletes and controls [23, 24].

Several echocardiography studies using techniques including tissue Doppler imaging and speckle tracking have been used to investigate left ventricular torsion in athletes. Some have reported similar findings to ours of decreased apical rotation and LV torsion in athletes with high levels of aerobic fitness [25] whereas others have reported that high intensity exercise either had no effect [26], or even lead to an increase [27] in LV torsion. The inconsistent results that have been reported may in part reflect different sport and training techniques, research methodology used and also the difficulty in positioning the apical and basal slices in echocardiography studies, which is based upon anatomical landmarks with a degree of subjectivity. In CMR on the other hand, positioning of the slices is carried out objectively based upon the length of the ventricle [17].

It has previously been suggested that decreased torsion in athletes is mediated by eccentric hypertrophy of the LV with decreased lever arm forces from epicardial fibres [28]. Athletes in the present study displayed eccentric hypertrophy (higher LVMI but LVM/EDV not significantly different than controls) despite this there was no significant association between twist or torsion and markers of remodelling such as LVM/EDV. Furthermore on linear regression analysis there was no association between LT and LVEDVI or LVMI. This suggests it is likely that the association between LT and decreased LV torsion is more complex than decreased lever arm forces.

The trend to correlation between LVMI and LV torsion may reflect that as athletes train they increase both V̇O2max and LT (although different athletes and training programmes increase these two parameters at different rates). The training done to increase V̇O2max tends to be associated with an increase in LV mass [14–16] and we have reported that a higher LT is associated with decreased torsion.

An alternative mechanism for decreased torsion in athletes with high LT is cellular changes within the heart as a consequence of high-intensity training. It is well recognised that LT particularly as a proportion of V̇O_2max_ is a key determinant of performance in endurance sport [29] and endurance athletes can increase their LT by regular high intensity training (such as interval training). Interval training has been associated with increased vascularity [30] and improved substrate utilisation [31] within the myocardium. It has also been reported that interval training leads to depressed LV ejection fraction, untwist rate, apical rotation rate and circumferential strain 30 min after a training session [23].

In diffuse myocardial diseases [32, 33] increased LV torsion is attributed to subendocardial microvascular hypoperfusion and contractile dysfunction. It is hypothesized that there is a compensatory increase in subepicardial fibre contraction giving rise to increased LV torsion yet unchanged overall circumferential strain. It is possible that the converse could be true in athletes with high intensity training leading to increased vascularity of the subendocardium. This would explain the correlation between LT and LV torsion but not measures of LV strain.

### The relationship between strain parameters and V̇O_2max_

We have also reported lower RV peak longitudinal strain, LV LDSR, RV EDSR and LDSR in athletes versus controls. On multivariable linear regression only increasing RV LDSR was associated with lower V̇O_2max_. Although this result is less striking, to our knowledge it is the first time that CMR strain techniques have been used to compare longitudinal functional changes in athletes and controls. Conflicting findings have been reported when echocardiographic assessment of diastolic function has been used with some studies reporting augmented relaxation of both ventricles in endurance athletes [4, 34] and others reporting no difference from controls [35]. Our finding of reduced longitudinal LDSR of both ventricles of athletes and a negative association between longitudinal RV LDSR and V̇O_2max_ can be attributed to reduced active atrial contraction late in diastole. In athletes the atrial contribution to ventricular filling is not required at rest but of course can then be utilised to maximise cardiac output during exercise.

### Limitations

Our study was carried out on a cross sectional cohort and it is important in the future to demonstrate that these findings can be replicated in a longitudinal study. The cohort in the study also has a wide range of age and athletic ability; although this was deliberate to allow study of athletes with a range of aerobic fitness. We have only studied the contribution of cardiac performance, and not ventilatory or neuromuscular function, to measures of aerobic fitness. All of the cardiac changes reported are during rest. Many of the strain parameters measured may not be independent and analysis of their individual interactions with aerobic fitness requires more complex modelling. We have not performed this in our study as very few strain parameters were associated with aerobic fitness.

## Conclusions

This cohort of endurance athletes had lower LV circumferential strain, LV torsion and biventricular diastolic strain rates than controls. Increased LT, which is a major determinant of performance in endurance athletes, was associated with decreased LV torsion. Further work is needed to understand the mechanisms by which this occurs.

## Abbreviations

CMR, cardiovascular magnetic resonance; CPX, cardiopulmonary exercise testing; EDV, end diastolic volume; EDVI, end diastolic volume indexed to body surface area; ESV, end systolic volume; LT, lactate threshold; LV, left ventricle; LVM, left ventricle mass; LVMI, left ventricle mass indexed to body surface area; RV, right ventricle; SPAMM, spatial modulation of magnetization; SSFP, steady state free procession; V̇O2max, maximal oxygen uptake (ml/min/kg)
